# The Principles of Antiseptic Surgery

**Published:** 1877-01

**Authors:** Jarvis S. Wight


### THE PRINCIPLES OF ANTISEPTIC SURGERY.

BY JARVIS S. WIGHT, M. D.

*Professor of Surgery and Clinical Surgery at the Long Island College Hospital.*

[ The speaker, after having explained the most recent views on the nature of the processes of fermentation and putrefaction, proceeded :—]

It appears, therefore, that the cells of nutrition absorb, decompose and eject some forms of matter, in a manner similar to torulæ, bacteria and septic germs. And this similarity elucidates the subject of antiseptics and disinfectants. But antiseptic surgery depends on infection and sepsis; for without infection there would be no antiseptic surgery ; and to understand antiseptic surgery implies a knowledge of antiseptics and disinfectants.

Let us at once define the terms antiseptic and disinfectants: —1. An antiseptic is a preventor of sepsis. For example, when carbolic acid preserves septic germs, so that they can not infect, it acts as an antisptic. And, 2. A disinfectant is an arrester of sepsis. For example, when chlorine decomposes septic products and septic germs, it acts as a disinfectant. In a general way, fermentation and putrefaction may be included in these definitions. So that a preventer of fermentation and putrefaction may be called an antiseptic; and an arrester of fermentation and putrefaction may be called a disinfectant. Hence any place containing fermentable or putrescible matter may be antisepted; and any place containing fermenting or putrefying matter may be disinfected. Notably, however, antiseptics prevent septic changes taking place in the body, that is, they arrest the changes caused by infection.

On the one hand there is a nutrition cell; on the other hand there is a septic germ; and the septic germ is ready to infect the nutrition cell. In one case it is possible to antisept, preserve, or disable the septic germ, so that it can not infect the nutrition cell; in another case the nutrition cell may be so changed by an antiseptic that it can not be infected by the septic germ; and in still another case it is competent to affect by an antiseptic both the septic germ and the nutrition cell, so that they can not act upon each other.

Now suppose that the nutrition cell is infected by the septic germ, that the bacterium is using up the oxygen of the cells, that sepsis is going on. Again, in one case the septic germ may be destroyed, so that infection ceases, that is, the nutrition cell is disinfected; in another case the nutrition cell may be destroyed, and then infection is arrested; and in still another case both the septic germ and the nutrition cell may be destroyed, and then sepsis can no longer go on.

In this connection two important observations may be made:—1. Locally, the infected nutrition cells may be destroyed either by an antiseptie or a disinfectant, so as to limit the sepsis, without harm to the general system. 2. If the sepsis is general, it must be arrested by the use of some agent that will not injure the nutrition cells, but will destroy the life of the septic germs.

Here, while admitting the importance of the germ theory, and the force of septic germs, I wish to state that there are many other forces surrounding and involving the living cells, which may, from time to time, become morbific in their tendency. These forces, such as gravity, heat and light, may, under favorable conditions, so far affect the cells of nutrition, that they, for a longer or shorter time, may live a mor bid life. And these cells during this morbid life, make waste products that are extremely offensive, and readily infected by septic germs; and then the morbid cells themselves will become infected, and finally the sepsis will invade the healthy neighboring cells. For instance, let me direct attention to the well nourished body; it develops, grows, moves, and works in the midst of forces, such as gravity, cohesion, affinity, heat and light, that is, its existence is intimately related to these forces. In fact, the harmonious, cooperation of these forces makes organization possible; and if this harmony be broken, disorganization supervenes. Precipitate the body from a sufficient height, and it is dashed in pieces; wrap it in flames, and it melts into ashes and dust; pour the sunbeams of summer on it, and it may perish; let the lightning strike it, and the poles of its organic affinity are torn asunder; and overdose it with potential drugs, and its vital motions cease.

Under the abnormal action of physical forces nutrition is impaired. The cells are injured by a fall, a blow, or a burn; they are not killed outright; they do not die at once; they try to live; they struggle on for a while; it is a morbid life; in fact, they are sick, some unto death and some unto life; it is slow work, but they at last recover; and while they are sick, they behave like ferments, putrefacients, septics and nutrifacients; they die perhaps, and enter the blood and lymph, which they contaminate and poison, and then poison the general nutrition; and the sick cells may go into the general circulation, and infect the healthy cells; and what is more, the waste products of this impaired nutrition may permeate the lymph spaces, the lymphatics, and the blood vessels, and disturb more or less seriously the nutrition; and then there supervenes a condition like sepsis, a sepsis without the septics; there is an infection without the bacteria, a contamination by sick cells and their waste products; but one says, there is surgical fever; and another says, there is inflammatory fever; there is a something which gets into the streams of the circulation, and affects the blood cells; this something and the contaminated blood cells go out into the tissues; there are wandering cells, repair cells, and pus cells; and these cells, in their diapedesis, are below par; they affect, if they do not infect, the cells of the tissues, and impair their nutrition. Yes, perhaps the fluids of the blood, the lymph, the tissues, the granulations, and the pus contain during surgical fever, and inflammatory fever the infection; and perhaps the corpuscular elements have no harm and no blame; and it may be that the infection is a chemical poison generated for a time, or it may be that these fluids are infested with bacteria.

Give the bacterium his due, for he is a scavenger, and may begin his work too soon, but that is not his fault, since he may mistake a dying cell for a dead one. But there are other factors besides bacteria that claim the attention of the antiseptic surgeon. The baneful effects of the products of gangrene and ulceration, and of the waste products of repair, have been too little considered. How careful we are to remove the waste products of nutrition; carbonic oxide, urea, excrement, and animal matters are borne away by the air or covered by the soil. Shall, then, granulations consume their own waste products? Shall the material used and ejected be used over again, as if it were fresh and pure? Ventilate wounds, amputations and ulcers, and wash away the waste products. But the objector says, you admit the mote-laden air; and this air is full of germs, diseases germs, septics, and you must keep away the air to exclude the germs. But, in reply, there are germs that live without oxygen, and the more the air is excluded the more these germs can flourish. Besides, the granulations must have oxygen, or they sicken, and then the anærobic germs can attack and feed on them. In the absence of complete proof, it is easy to assume the universality of air germs. Germs are, perhaps, well nigh universal; but only some of them are disease germs. It is at least admitted that the bacteria are concomitants of disease, while the infusoria are generally quite harmless. If every germ of the air were a desease germ, surely it would be a wonder if the germs were not the survivors of the nutrition cells, and thus the "fittest," and then the race would come to an end.

It appears, therefore, that antiseptic surgery is obliged to deal with the antagonism between different kinds of living matter. It seeks the best means of preventing and arresting the invasion of the tissue cells by septic germs; for the prevention of infection suggests the subject of disinfection. And a substances in one relatien may be an antiseptic, and in another relation may be a disinfectant. For instance, carbolic acid, when applied to an infected wound, may antisept the the infecting virus, and disinfect the wound; but if chlorine be diffused through an infected hospital ward, it will destroy the infecting virus, disinfecting the wards and the various objects that it contains.

Antiseptic surgery has become famous, so has carbolic acid; and the fame of carbolic acid comes from its antiseptic power. But carbolic acid has other properties; carbolic acid is an astringent, arresting bleeding from small vessels, and preventing the entrance of septic germs into the lymph spaces, lymphatics, and blood capillaries; it is a local anodyne, alleviating the pain and the shock of injury; it is a local anæsthetic, mitigating and preventing the pain and shock of operations; it slows, or inhibits the amœboid motion of the white cells, often modifying in a favorable manner the process of plastic infiltration; it diminishes reflex irritation, controlling the action of the vaso-motor nerves, limiting congestion and exudation, and tending to subdue inflammation. See how many substantial reasons why carbolic acid may be used in surgery; and these reasons are not because carbolic acid is antiseptic. Please to remember that I do not ignore the antiseptic property of carbolic acid. I simply object to the apotheosis of its antisepsis. That it can from time to time, prevent and modify infection and sepsis is a great gain to surgical therapeutics; but it can also kill and mummify tissue cells, white cells, repair cells, granulation cells, and pus cells. I am unable to say whether it will kill the septic germs or the cells of nutrition first. I do not know what form of living matter can longest resist the antiseptic action of carbolic acid. One thing is pretty evident, the internal use of carbolic acid can do but little good in general infection and sepsis, for, if it destroys the so-called septic germs, it may also destroy the cells of nutrition. And should there be a sepsis, or a fermentation, or a zymosis, by the living cells of the blood and tissues, carbolic acid as an antiseptic would certainly not be indicated, especially when we possess such a remedy as quinia. The theory and the practice of some of the advocates of antiseptic surgery may be put into the form of a syllogism. The mote-laden air is full of germs; germs are the cause of septicæmia; carbolic acid will destroy septic germ; therefore, carbolic acid will prevent septicæmia. The air is full of germs, we admit, and the air germs may be the cause of sepsis, we also admit; but septic conditions arise when there are no germs except the sick cells; while many of the air germs are innocuous; and even if carbolic acid, most vigourously applied, will prevent septicæmia, it can not prevent the "septoid" work of injured and sick nutrition cells. So the syllogism does not contain all the facts of the problem; and if it did, the completeness of the practical work that it proposes would be simply impossible.

To turn from this picture of putting carbolie acid on the septic germ, I have said that there are anaerobic germs; they are, perhaps, the vibrios, so called; they live in the depths of some fluid containing putrescible matter; they, like fish can not live in the air; they go into the circulation and penetrate the tissues; they liberate the sulphur, phosphorus and nitrogen, which combine with hydrogen and go into the air in the form of offensive gases that are slowly oxidized. This disastrous work of the vibrios, going on after death, may begin during life, may originate in the waste products of nutrition or repair, and eventuate in the decomposition of the living cells. But a beautiful provision of nature comes in here to our aid. A disinfectant, of undoubted power, too little understood, too little appreciated, and not used as much as it ought to be, can do two important things. I mean chlorine, which has a strong affinity for hydrogen. Chlorine will abstract hydrogen from its compounds with sulphur, phosphorus and nitrogen, and this abstraction of hydrogen destroys septic and putrid products, which, as every one knows, act as powerful chemical poisons, Chlorine will take hydrogen from water, and leave nascent oxygen, which is like ozone, and will oxidize the bacteria and kill the vibrios, Chlorine decomposes septic products and destroys septic germs.

The vast practical importance of this subject can not better be illustrated than by going over the main points in the history of an outbreak of septicæmia in the Long Island College Hospital, during the spring and summer of 1873. The medical officers of the Executive Committee requested me to submit a plan for purifying the infected wards. The following plan, approved by my colleagues, was carried out, under my personal supervision. The patients and everything movable, except the bedstead, were removed from the infected wards. The straw, some of the bedding, and some of the clothing were burned; the rest of the bedding and the clothing were put into a strong solution of carbolic acid for two days and then taken out, when they were washed and dried; the wards containing the bedsteads were fumigated, for three days, with the hot vapor of carbolic acid, when the walls, floors and bedsteads were scrubbed with a strong solution of carbolic acid; everything was then thoroughly ventilated, and the septic demon went on with the same pace as before the lavish expenditure of carbolic acid. So much that time for the use and fame of the great antiseptic. We were powerless, or in error; we shall see.

Later on in the season I came on duty in the surgical wards, our fruitless work of purification still fresh in memory. The results had been bad, and the prospects were discouraging. Could anything be done to arrest and avert this sepsis, this infection, and banish the septic germs? It then occurred to me that I had used an antiseptic which might preserve the septic germs for a time, and afterward evaporate, leaving the germs alive and ready to continue their work. Surely I had overlooked the surgeon, the interne, and the nurse, whose hands might be the nursery for all kinds of septic germs, ready for transplantation into the first congenial soil, and the waste products of nutrition and repair would constitute such soil, and are abundant in the wards of a hospital. Remembering the disinfecting power of chlorine, I determined to use it, which I did in the following manner;—The hands of the surgeon, the interne, and the nurse, and all instruments and appliances, were disinfected by a solution of chlorinated soda, before each operation and each dressing of a wound, and in about two weeks sepsis had ceased its ravages. During this time, and subsequently, some wounds were dressed with carbolized oil, some with sweet oil, some with castor oil, some with a solution of chlorinated soda, some with zinc ointment, some with red wash, some with fresh water, and some with dry oakum, and there was no return of the sepsis, nor has there been to this day; but a bottle of liquor sodæ chlorinatæ has been standing on the table in each of the wards for constant use ever since.

Suppose the germ theory to be true—for it is good enough to be true—it is not universal; all diseases are not due to septic germs, though some are, and the destruction of septic germs before they infect prevents sepsis and disease. Admit that antisepsis is good practice, it must be further admitted that disinfection of infecting material is better practice, for it decomposes, where antisepsis might preserve. And this better practice may be illustrated and proved by experience. At any rate, the most rigid antiseptic surgery, so-called, may be equaled by careful disinfectation and ventilation. Clean hands, clean air, clean water, clean sponges, clean instruments, clean bedding, clean wards, clean patients, and clean wounds, by whatever means obtained, are the ground work for the best results. And it is true, that, in Scotland, Germany, England and America, cleanliness has done as much, if not more, than the great antiseptic, for the safety and recovery of surgical patients. Cleanliness has been overlooked, and the pleasing results of a beautiful theory and a magnificent practice, which are the post hoc, have been looked upon as the propter hoc of carbolic acid. I do not condemn carbolic acid, but use it constantly, for it is a priceless remedy, having wonderful powers, such as were unknown when it was first employed in the healing art.

I have spoken of the waste products of repair; I mean the repair of injury. It is true that nature is prodigal of material; this is especially so of repair; much more than is needed is furnished, and the surplus is or ought to be removed. Can the ferments live on alcohol? Can the bacteria live on sulphuretted hydrogen? Can the liver cells live on bile? Can the blood cells live on carbonic acid? Can the vibrios live on air? And can the granulation cells live on pus? Surely it would not be good practice to compel the cells of repair to consume their own waste products, or to turn these products back into the general system to poison it.

It is not my purpose here to give a lengthy consideration of the various antiseptics and disinfectants. I simply note some of them. Pulverized charcoal, whose carbon atoms attract and condense the oxygen atoms of the air into an active agent like ozone; permanganate of potassa, whose abundant oxygen is liberated, and becomes nascent and active; and sulphurous acid, which also parts with oxygen, rendering it like ozone, may, on account of this condensed and active oxygen, superoxidize the bacteria, and oxidize the vibrios, and put an end to sepsis, as well as putrefaction and fermentation. I might dwell on the use of quinia, which has the extraordinary power of destroying some kinds of septic germs as they are at work amid the living cells, and inhibiting the activity of some others, when the nutrition cells can not alone withstand them; and I might rehearse the good qualities of alcohol as an incompatible to septic germs, and a sustainer of nutrition cells, but I forbear as my object was to make some remarks on sepsis and antiseptic surgery.

And, in conclusion, in doing this, I have traced the thread that runs through living matter in its various forms. I have compared, in a certain way, sepsis with fermentation, putrefaction and nutrition, and have also compared impaired nutrition, caused by the abnormal action of physical forces, to sepsis, showing the limited application of the germ theory to the practice of antiseptic surgery; and I have intimated that after all sepsis may be a kind of putrefaction, modified and changed by the resisting and yet impaired action of living cell; a putrefaction beginning, under certain conditions and relations, in the waste products of nutrition and repair, before these products are removed from the vicinity of the living cells that formed them; in fine, that there is a contest betwixt nutrition and putrefaction, a battle of the pigmies; a mistake, perhaps, of the putrefactients in the matter that they are called upon providentially to decompose for vegetation; flesh that can not or ought not to live; and so the scavengers come early, bent on destroying the putrescible individual, but, in the main, wholesome to the many who live; and I have not denied the value of preserving the scavenger until other natural causes may have done his work; but I have said that the destruction of the initial soil of infection, and the consequent destruction of the septic germ, is the best method of antiseptic surgery. The principle involved here seems to be sound. In essentials there should be agreement; while in particulars, leading to the essentials, there may be a difference of opinion and a variety of practice.

